# Resistance to Ag(I) Cations in Bacteria: Environments, Genes and Proteins

**DOI:** 10.1155/MBD.1999.315

**Published:** 1999

**Authors:** Simon Silver, Amit Gupta, Kazuaki Matsui, Jeng-Fan Lo

**Affiliations:** Department of Microbiology and Immunology University of Illinois Chicago IL 60612-7340 USA

## Abstract

Bacterial resistance to Ag(I) has been reported periodically with isolates from many environments where toxic levels of silver might be expected to occur, but initial reports were limited to the occurrence of resistant bacteria. The availability of silver-resistance conferring DNA sequences now allow genetic and mechanistic studies that had basically been missing. The genes determining Ag(I) resistance were sequenced from a plasmid found in a burn ward isolate. The 14.2 kb determinant contains seven recognized genes, arranged in three mRNA transcriptional units. The *silE* gene determines an extracellular (periplasmic space) metal-binding protein of 123 amino acids, including ten histidine residues implicated in Ag(I) binding. SilE is homologous to PcoE, of copper resistance. The next two genes, *silR* and *silS*, determine a two protein, histidine-kinase membrane sensor and aspartyl phosphate transcriptional responder, similar to other two component systems such as CzcR and CzcS (for cadmium, zinc and cobalt resistance) and PcoR and PcoS (for copper resistance). The remaining four genes, *silCBAP*, are co-transcribed and appear to determine Ag^+^ efflux, with SilCBA homologous to CzcCBA, a three component cation/proton antiporter, and SilP a novel P-type ATPase with a amino-terminal histidine-rich cation-specificity region. The effects of increasing Ag^+^ concentrations and growth medium halides (Cl-, Br- and I-) have been characterized, with lower Cl- concentrations facilitating resistance and higher concentrations toxicity. The properties of this unique Ag(I)-binding SilE protein are being characterized. Sequences similar to the silver-resistance DNA are being characterized by Southern blot DNA/DNA hybridization, PCR *in vitro* DNA synthesis and DNA sequencing. More than 25 additional closely related sequences have been identified in bacteria from diverse sources. Initial DNA sequencing results shows approximately 5-20% differences in DNA sequences.


### RESISTANCE TO Ag(I) CATIONS IN BACTERIA: ENVIRONMENTS, GENES AND PROTEINS

Simon Silver*, Amit Gupta, Kazuaki Matsui, and Jeng-Fan Lo

Department of Microbiology and Immunology, University of Illinois, Chicago, IL 60612-7340, USA

### ABSTRACT
Bacterial resistance to Ag(I) has been reported periodically with isolates from many environments where toxic levels of silver might be expected to occur, but initial reports were limited to the occurrence of resistant bacteria. The availability of silver-resistance conferring DNA sequences now allow genetic and mechanistic studies that had basically been missing. The genes determining Ag(I) resistance were sequenced from a plasmid found in a burn ward isolate. The 14.2 kb determinant contains seven recognized genes, arranged in three mRNA transcriptional units. The *silE* gene determines an extracellular (periplasmic space) metal-binding protein of 123 amino acids, including ten histidine residues implicated in Ag(I) binding. SilE is homologous to PcoE, of copper resistance. The next two genes, *silR* and *silS,* determine a two protein, histidine-kinase membrane sensor and aspartyl phosphate transcriptional responder, similar to other two component systems such as CzcR and CzcS (for cadmium, zinc and cobalt resistance) and PcoR and PcoS (for copper resistance). The remaining four genes, *silCBAP,* are co-transcribed and appear to determine $Ag^+$ efflux, with SilCBA homologous to CzcCBA, a three component cation/proton antiporter, and SilP a novel P-type ATPase with a amino-terminal histidine-rich cation-specificity region. The effects of increasing $Ag^+$ concentrations and growth medium halides ($Cl^-$, $Br^-$ and $I^-$) have been characterized, with lower $Cl^-$ concentrations facilitating resistance and higher concentrations toxicity. The properties of this unique Ag(I)-binding SilE protein are being characterized. Sequences similar to the silver-resistance DNA are being characterized by Southern blot DNA/DNA hybridization, PCR *in vitro* DNA synthesis and DNA sequencing. More than 25 additional closely related sequences have been identified in bacteria from diverse sources. Initial DNA sequencing results shows approximately 5-20% differences in DNA sequences.

### INTRODUCTION
The mechanisms of resistance to heavy metals that are encoded by various plasmid-based genes have been thoroughly studied.[1,2] The best understood such system is that for resistance to inorganic mercury and organomercurials.[1-3] The basis of mercury resistance is enzymatic cleavage of the Hg-C bond of organomercurials to release $Hg^{2+}$, followed by reduction of $Hg^{2+}$ to volatile $Hg^0$ by the dimeric protein mercuric reductase,[3] for which one example has a structure solved by x-ray crystallography.[4] This x-ray derived structure of mercuric reductase plus the crystal structure of a cadmium-responding transcriptional repressor protein[5] and the NMR solution of a periplasmic mercury binding protein[6] are the only available protein structures for the several dozen different proteins involved in various metal ion resistance systems.[1,2] A related copper- and silver-binding domain of a human protein has been solved.[7] The bacterial mercury and human copper binding domains are basically similar in secondary and tertiary folding, as expected, and specific yet-to-be understood differences in the amino acids surrounding the binding site must account for the different binding specificities. Most toxic heavy metal resistances result not from chemical detoxification, but from energy-dependent "pumping out" of the toxic metal ion from the cell by membrane proteins that function either as ATPases or as chemiosmotic cation/proton antiporters.[1,2] Ag(I) resistance becomes the newest example of such efflux pumping.

Silver resistant bacteria have been found repeatedly in environments where silver toxicity might be expected to select for resistance, such as burn wards of hospitals where silver salts (silver nitrate but especially silver sulfadiazine) are used as antiseptics to treat burns.[8-11] The wide variety of other environments where silver is found and/or used has recently been reviewed.[12,13,14] These include clinical use of silver or silver-coated catheters,[15-18] soil around mines,[19,20] catchment areas associated with photographic film production and processing,[21] and water distribution systems where metal compounds are used for control of infectious agents, such as *Legionella.*[22] The most common human exposure to Ag is with dental amalgams that contain 50% Ag;[23] since the other major component of amalgams, Hg(0), is slowly released in to the body.[24] Since it is clear that released Hg(0) is oxidized and then selects for Hg-resistant bacteria,[24] it seems likely that a similar release of Ag(0) followed by oxidation to Ag(I) occurs but has never been measured. Silver-containing consumer products include silver-coated mints in Japan, $Ag^{+}$-citrate complexes as health food additives in Florida, domestic water purification cartridges in the USA, and super market-available colloidal silver for washing salad vegetables in Mexico. Silver is familiar in laboratory use as a stain for proteins in polyacrylamide gels. There are uncertainties about the details, but the silver in stained gels is reduced polymeric Ag(0).[25] The staining process involves several stages. Initially Ag(I) binds to previously denatured protein, a process dominated by histidine residues. This is followed by stabilization of the polymeric Ag-center, where an initial reduction event is catalyzed and then nucleates multiple reduction events.[25]

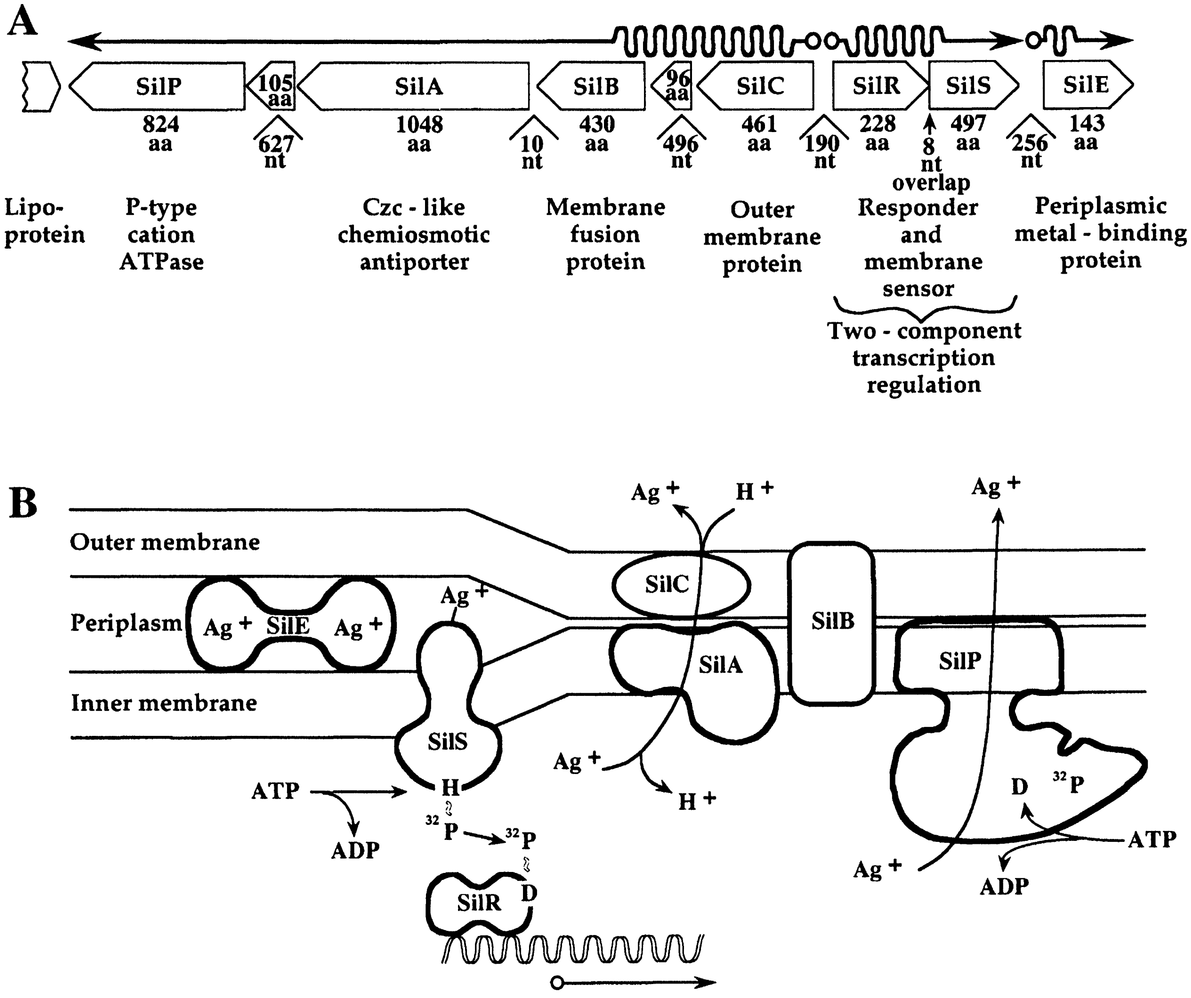

**Figure 1.** Silver resistance genes, transcripts and protein products. A, Top line shows the mRNAs. The open boxes indicate different genes or open reading frames (ORFs) and their orientations. Nucleotides (nt) between genes and the sizes of gene products in amino acids (aa) are marked. B, The proposed function of each gene product, deduced from homologies to known proteins.[27, with permission]

### RESULTS

Plasmid pMG101[26] is a 180 kb silver resistance plasmid[27] that also confers resistances to $Hg^{2+}$ and tellurite, and to several antibiotics. Fragments of pMG101 were cloned and transformants screened for increased resistance to $Ag^{+}$.[27] The use of several clones and primer walking led to 14.2 kb of sequence (GenBank accession # AF067954[27]) with seven recognized genes. On the right of the silver resistance determinant (Fig. 1A), the first gene, *silE,* determines a small periplasmic protein that is 47% identical to PcoE, of the *E. coli* plasmid copper resistance system.[28] The SilE

polypeptide has its first 20 amino acids removed on movement across the membrane to the periplasm and is synthesized only during growth in the presence of $Ag^+$.[27] *silE* and *pcoE* DNAs and upstream transcriptional promoters and regulatory sites are homologous;[27,28,29] but the sequences downstream of silE and pcoE are unrelated.

Upstream from *silE* are the *silRS* genes for a presumed two component signal transduction pair, consisting of a membrane kinase sensor and transcriptional regulatory responder, homologous to other two component family pairs.[30] The deduced SilRS sequences are most closely related to a new sensor/responder pair that was identified in the final segment of the *E. coli* chromosome sequence to be sequenced.[31] The copper resistance Pco system includes *pcoRS* upstream of *pcoE*.[28]

Upstream of *silRS*, the orientation of the genes and the nature of the gene products of the silver resistance system are unrelated to the *pco* copper resistance system. The six open reading frames (ORF; that is potential genes which would encode proposed proteins that would be responsible for the resistance) in the silver resistance system are transcribed divergently from *silRSE* (Fig. 1A). The *silCBA* genes appear to determine a three-component, membrane potential-dependent, cation/proton antiporter,[32,33,34] with homologs in the cadmium, zinc and cobalt resistance system (Czc) of *Alcaligenes.* The components of this presumed $Ag^+$ efflux system are the inner membrane proton/cation antiporter, SilA, the membrane fusion protein, SilB, that brings together the inner and outer membranes of Gram negative bacteria,[34,35] and the outer membrane protein, SilC. Between *silC* and *silB*, a small ORF was identified (Fig. 1A) that could determine a polypeptide of 96 amino acids, which would be 45% identical to the product of a 110 amino acid long ORF in the *E. coli* chromosomal homolog.[31] This ORF of unknown function is not found in other homologous CBA systems.[33,34,35] Another ORF, potentially encoding a protein of 105 amino acids in length, was found between *silA* and *silP* (Fig. 1A), but the product of this ORF lacks known homologs.[27]

**Table 1**. Analysis of metal binding by SilE protein by ICP[a]

| Protein sample | Metal content gatoms/mol protein Ag | Cu | Cd |
|---|---|---|---|
| protein at 76 μM, pH 5 | | | |
| Apo-protein | ≤ 0.01 | ≤ 0.01 | ≤ 0.01 |
| Ag-loaded 1 mM | 10.8 | ≤ 0.01 | ≤ 0.01 |
| Cu-loaded 1 mM | ≤ 0.01 | ≤ 0.01 | ≤ 0.01 |
| Cd-loaded 1 mM | ≤ 0.01 | ≤ 0.01 | ≤ 0.01 |
| protein at 10 μM, pH 5 | | | |
| Ag-loaded 10 μM | 0.02 | ≤ 0.01 | ≤ 0.01 |
| 1 mM | 38.2 | ≤ 0.01 | ≤ 0.01 |
| Cu-loaded 1 mM | ≤ 0.01 | ≤ 0.01 | ≤ 0.01 |
| protein at 10 μM, pH 7.5 | | | |
| Ag-loaded 10 μM | 1.1 | ≤ 0.01 | ≤ 0.01 |
| 1 mM | 5.5 | ≤ 0.01 | ≤ 0.01 |
| Cu-loaded 1 mM | ≤ 0.2 | ≤ 0.01 | ≤ 0.01 |

[a]Purified SilE protein was exposed overnight in buffer to $Ag^+$, $Cu^{2+}$ or $Cd^{2+}$, and after exhaustive dialysis against metal-free buffer, the protein and metal content were measured. No other metal ions were detected by ICP analysis in these samples.

The deduced product of the final gene of the silver resistance determinant, on the left of Fig. 1A, is a 824 amino acid P-type ATPase, SilP. A deletion of DNA in the middle of *silP* results in reduced silver resistance by the transformed cells (K. Matsui, unpublished data). SilP belongs in the family of heavy-metal resistance efflux ATPases.[1,36] The SilP sequence contains all the specific features of this group of P-type ATPases, including the conserved region around the phosphorylated aspartyl residue, the ATP-binding region, the aspartyl-phosphatase determinant, the CysProCys conserved in the predicted sixth transmembrane alpha helical region (thought to be part of the cation translocation pathway), and the HisPro between the phosphorylation site, and the

ATP binding region in the large aspartyl kinase domain.[1,36] There is one striking difference between deduced SilP and earlier-described soft metal-efflux ATPases.[1,36] The previously-described cadmium, copper[7] or zinc efflux ATPases (in animals and bacteria) have sequences including GlyMetXCysXXCys towards the N-terminus and apparently in the cytoplasmic region.[1,2,36] These sequences are thought to provide cation binding for transport or its modulation.[37] SilP lacks cysteines in this location but has a $His_5AspHis_2$ instead in the comparable position. There is no *silP* homolog in the *E. coli* chromosome close to the *silABCRS* homologs. However, two histidine-rich P-type ATPase genes have been identified elsewhere on the *E. coli* chromosome.[38] There is no information concerning the cation specificity or physiological function of these ATPases.

The small periplasmic protein, SilE, was purified to homogeneity from bacterial periplasmic proteins, and its sequence confirmed by N-terminal amino acid sequencing (J.F. Lo, in preparation). Preliminary studies with purified SilE protein using atomic absorption spectroscopy (AAS) and inductive-coupled plasma (ICP) analysis showed very high specificity for Ag(I)-binding (Table 1). SilE protein that had not been loaded with metal ions (apo-SilE) contained less than one cation per 100 polypeptide chains (Table 1). When SilE protein was loaded with Ag(I), Cu(II) or Cd(II) and dialyzed, about 10 Ag(I) cations were retained per polypeptide, but less than one Cu(II) or Cd(II) per 100 polypeptide chains. The amount of Ag(I) bound depended on the Ag(I)/protein ratio, the pH during incubation, and whether Ag(I) was bound under concentrated (excess metal) conditions followed by dialysis away from the protein, or by adding dilute Ag(I) solutions outside the tube and dialysis inwards to the protein. In the absence of Ag(I), preliminary evidence for Cu-N bonding with SilE was obtained by electron spin resonance (ESR) spectroscopy (J.F. Lo, in preparation). Circular dichroism (CD) analysis of the SilE protein with and without added Ag(I) (J.F. Lo, in preparation) showed a striking change in spectrum from predominantly unstructured to mostly alpha helix on binding Ag(I). Folding to a well-defined structure on binding metal cations also occurs with the unrelated poly-cysteine protein metallothionein.[39]

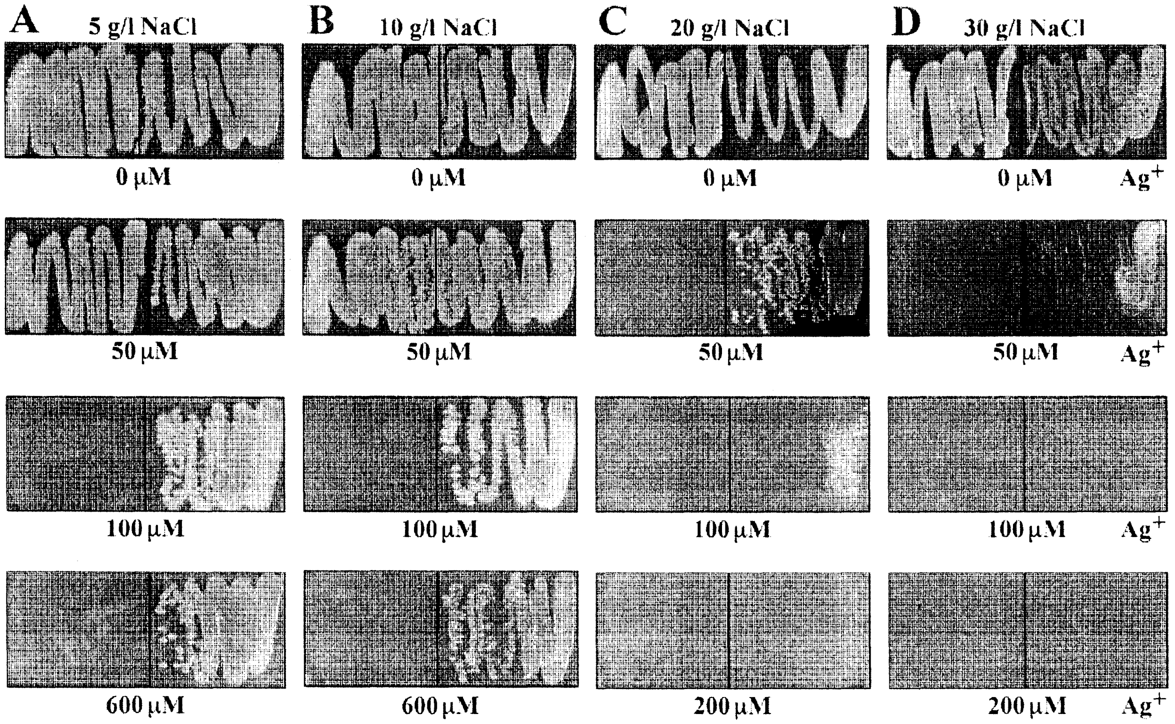

**Figure 2**. Growth of sensitive and resistant *E. coli* on LB broth (tryptone, peptone and yeast extract) with varying NaCl and Ag(I). Petri dishes containing LB broth agar with 5 to 30 g/l of NaCl (0.09 to 0.5 M) and 0 to 600 μM $Ag^+$ was streaked with a culture of sensitive (to the left of each section) or resistant (to the right) bacteria and surface growth after 20 h at 37°C photographed.[from 44 with permission]

Transcription of the silver resistance determinant was measured by Reverse Transcriptase-Polymerase Chain Reaction (RT-PCR), Northern blot RNA/DNA hybridization and primer extension analysis.[27] Three mRNAs are synthesized, one each for *silE*, *silRS* and *silCBAP*, as indicated in Fig. 1A. Their inducibility (by $Ag^+$) and precise start sites were determined.[2]

The *E. coli* chromosome has an uncharacterized six gene region (xxx*ABORF110CRS)* (listed in GenBank accession no. AE000162), that is more closely homologous to the new plasmid silver resistance *silABORF96CRS* than to other available sequences. The cation substrate for this presumed regulated cation efflux system is not known, but the candidates are $Ag^+$ (since mutations to silver resistance and $Ag^+$ efflux have been found with *E. coli*[40]) and $Cu^+$ (since a $Cu^+/Ag^+$ efflux ATPase has been identified in a different bacterial type[41]).

Conditions for distinguishing silver resistant from silver sensitive bacteria are not well-established, and even the existence of silver-resistant bacteria that cause a clinical problem has been challenged (A.T. McManus, the presentation immediately preceding this one at the ACS Symposium). It was useful to clarify some of the factors involved, in particular, halides ions can act as precipitating agents,[42] while proteins and other biological Ag(I)-ligands profoundly affect the "bioavailability" of Ag(I). Earlier and recent experiments[43,44] suggest three levels of effects: Firstly at low halide (chloride), especially in natural environments and in the clinic, soluble $Ag^+$ binds tightly to the bacterial cell surface, inhibiting respiration and having other toxic effects.[45,,46,47] Paradoxically, moderate levels of $Cl^-$ increase the Ag(I)-resistance of resistant bacteria while increasing the sensitivity of sensitive bacteria, perhaps by making Ag(I) more "bioavailable".[44] $Br^-$ has a similar effect to $Cl^-$, but functions at lower levels reflecting the lesser solubility of AgBr compared with AgCl.[42] And $I^-$ basically removes Ag(I) into a non-bioavailable precipitate.[44]

It is less familiar that Ag(I)-halide precipitates come back into solution at higher halide concentrations, readily forming water soluble anionic complexes ($AgX_2^-$ and $AgX_3^{2-}$), with relative stabilities $I^->Br^->Cl^-$.[48] The water soluble anionic Ag-halide complexes appear to make the Ag(I) more bioavailable, as high halide levels increase Ag(I) toxicity to both sensitive and resistant bacteria (Fig. 2).[26] Some results leading to these three conclusions are shown in Figure 2, where each section shows the growth on an agar surface of sensitive (to the left) and resistant bacteria (to the right). At 5 or 10 g/l NaCl, the sensitive bacteria stopped growing between 50 and 100 μM Ag(I). The resistant bacteria grow above 600 μM Ag(I). However, increased NaCl resulted in increased Ag(I) sensitivity for both sensitive and resistant bacteria (Fig. 2). In a colored photograph of Fig. 2, the bacterial growth appears slightly creamy in color, and the growth of the resistant cells on higher Ag(I) concentrations show black pigmentation that is likely to be due to reduced metallic Ag(0). Silver reduction does not occur during growth and therefore reduction is not the basis for resistance. This is an important conclusion.

Following the availability of the DNA sequence in Fig. 1,[27] we tested whether additional bacteria might be found with identical or similar Ag(I) resistance determinants and DNA sequences. Surprisingly, it is more difficult to measure silver resistance (Fig. 2)[44] than DNA homologies. The preliminary results to date (A. Gupta et al., in preparation) indicate that homologous DNA sequences can be identified by Southern blotting (DNA/DNA hybridization) and polymerase chain reaction (PCR; *in vitro* DNA synthesis) analysis and are found in many hospital isolates of a wide range of bacterial species.

A one-page summary of this new work recently appeared.[49]

### ACKNOWLEDGMENTS

We give thanks Anne O. Summers for the plasmid used to start this work, to Carlos Cervantes, Yolanda Fuchs, Anne Hendry, X.-Z. Li, and Paul Schrenkenberger for additional bacterial isolates, to Francisco Roberto and Paige Goodlove for sequencing, to William Antholine, Peter Gettins, Ed Huff, Bao-Shiang Bob Lee, and Frank Shaw for protein analysis, and to William Hendrickson, Dietrich Nies, Frank Shaw and William Walden for useful discussions. This work was supported by a grant from the Department of Energy.

### REFERENCES

1. Silver, S. & Phung, L.T. *Annu. Rev. Microbiol.* 50, 753-789 (1996).
2. Silver, S. *J. Industr. Microbiol. Biotech.* 20, 1-12 (1998).
3. Hobman, J.L. & Brown, N.L. in *Metal Ions in Biological Systems* vol 34, Sigel, A. & Sigel, H., Eds., Marcel Dekker, New York, pp 527-568 (1997).
4. Schiering, N., Kabsch, W., Moore, M.J, Distefano, M.D., Walsh, C.T. & Pai, E.F. *Nature* 352, 168-171 (1991).
5. Cook, W.J., Kar, S.R., Taylor, K.B. & Hall, L.M. *J. Mol. Biol.* 275, 337-346 (1998).

6. Steele, R.A. & Opella, S.J. *Biochemistry* 36, 6885-6895 (1997).
7. Gitschier, J., Moffat, B., Reilly, D., Wood, W.I. & Fairbrother, W.J. *Nature Struct. Biol.* 5, 47-54 (1998).
8. George, N., Faoagali, J. & Muller, M. *Burns* 23, 493-495 (1997).
9. Modak, S.M., Sampath, L. & Fox, C.L. Jr. *J. Burn Care Rehabil.* 9, 359-363 (1988).
10. Pruitt, B.A., Jr., McManus, A.T., Kim, S.H. & Goodwin, C.W. *World J. Surg.* 22, 135-145 (1998).
11. Chu, C.S., McManus, A.T., Matylevich, N.P., Mason, A.D. Jr, & Pruitt, B.A. Jr. *J. Trauma* 39, 273-278 (1995).
12. Russell, A.D. & Hugo, W.B. *Progress Med. Chem.* 31, 351-370 (1994).
13. Clement, J.L. & Jarrett, P.S. *Metal-based Drugs* 1, 467-482 (1994).
14. Slawson, R.M., Van Dyke, M.I., Lee, H. & Trevors, J.T. *Plasmid* 27, 72-79 (1992).
15. Sampath, L.A., Chowdhury, N., Caraos, L. & Modak, S.M. *J. Hosp. Infect.* 30, 201-210 (1995).
16. Gatter, N., Kohnen, W. & Jansen, B. *Zentralbl. Bakteriol.* 287, 157-169 (1998).
17. Greenfeld, J.I., Sampath, L., Popilskis, S.J., Brunnert, S.R., Stylianos, S. & Modak, S. *Crit. Care Med.* 23, 894-900 (1995).
18. Gabriel, M.M., Mayo, M.S., May, L.L., Simmons, R.B. & Ahearn, D.G. *Curr. Microbiol.* 33, 1-5 (1996).
19. Haefeli, C., Franklin, C. & Hardy, K. *J. Bacteriol.* 158, 389-392 (1984).
20. Slawson, R.M., Lohmeier-Vogel, E.M., Lee, H. & Trevors, J.T. *Biometals* 7, 30-40 (1994).
21. Belly, R.T. & Kydd, G.C. *Dev. Indust. Microbiol.* 23, 567-577 (1982).
22. Liu, Z., Stout, J.E., Boldin, M., Rugh, J., Diven, W. F & Lu, V.L. *Clin. Infect. Dis.* 26, 138-140 (1998).
23. Dunne, S.M. & Gainsford, I.D. *Internat. Dental J.* 47, 123-136 (1997).
24. Lorscheider, F.L., Vimy, M.J. & Summers, A.O. *FASEB J.* 9, 504-508 and 1499-1500 (1995).
25. Heukeshoven, J. & Dernick, R. *Electrophoresis* 6, 103-112 (1985).
26. McHugh, S.L., Moellering, R.C., Hopkins, C.C. & Swartz, M.N. *Lancet* i, 235-240 (1975).
27. Gupta, A., Matsui, K., Lo, J.-F. & Silver, S. *Nature Medicine,* in press (1998).
28. Brown, N.L., Barrett, S.R., Camakaris, J., Lee, B.T.O. & Rouch, D.A. *Mol. Microbiol.* 17, 1153-1166(1995).
29. Rouch, D.A. & Brown, N.L. *Microbiol.* 143, 1191-1202 (1997).
30. Hoch, J.A. & Silhavy, T.J., Eds. *Two-Component Signal Transduction*, ASM Press, Washington, DC (1995).
31. Blattner, F. R. *et al. Science* 277, 1453-1474 (1997).
32. Nies, D.H. *J. Bacteriol.* 177, 2707-2712 (1995).
33. Nies, D. H. & Silver, S. *J. Indust. Microbiol.* 14, 186-199 (1995).
34. Nies, D.H. & Brown, N.L., in *Metal Ions in Gene Regulation,* Silver, S. & Walden, W., Eds., Chapman and Hall, New York, pp 77-103 (1997).
35. Ji, G. & Silver, S. *J. Indust. Microbiol.* 14, 61-75 (1995).
36. Solioz, M. & Vulpe, C. *Trends Biochem. Sci.* 21, 237-241 (1996).
37. DiDonato, M., Narindrasorasak, S., Forbes, J.R. , Cox, D.W. & Sarkar, B. *J. Biol. Chem.* 272, 33279-33282 (1997).
38. Trenor III, C., Lin, W. & Andrews, N.C. *Biochem. Biophys. Res. Commun.* 205, 1644-1650 (1994).
39. Zelazowski, A.J., Gasyna, Z. & Stillman, M.J. *J. Biol. Chem.* 264, 17091-17099 (1989).
40. Li, X.Z., Nikaido, H. & Williams, K.E. *J. Bacteriol.* 179, 6127-6132 (1997).
41. Solioz, M. & Odermatt, A. *J. Biol. Chem.* 270, 9217-9221 (1995).
42. Weast, R. C., Ed., in *CRC Handbook of Chemistry and Physics, $49^{th}$ edition.* The Chemical Rubber Company, Cleveland OH, p B-307 (1968).
43. Silver, S., in *Molecular Biology, Pathogenicity and Ecology of Bacterial Plasmids,* Levy, S.B., Clowes, R.C.& Koenig, E.L., Eds., Plenum Press, New York, pp 179-189 (1981).
44. Gupta, A., Maynes, M. & Silver, S. *Appl. Environ. Microbiol.* 64(12), in press, December issue, page number will be added (1998).
45. Schreurs, W.J.A. & Rosenberg, H. *J. Bacteriol.* 152, 7-13 (1982).
46. Bragg, P.D. & Rainne, D.G. *Canad. J. Microbiol.* 20, 883-889 (1974).
47. Ghandour, W., Hubbard, J.A., Deistung J., Hughes, M.N. & Poole, R.K. *Appl. Microbiol. Biotech.* 28, 559-565 (1988).
48. Cotton, F.A. & Wilkinson, G., in *Advanced Inorganic Chemistry, 5th Edition,* John Wiley and Sons, Inc., New York , pp 937-949 (1988).
49. Gupta, A. & Silver, S. *Nature Biotechnology* 16, 888 (1998).

